# Connecting Molecular Characteristics of Intrauterine Growth-Retarded Piglets to Targeted Nutritional Interventions: A Review

**DOI:** 10.3390/ani15152231

**Published:** 2025-07-29

**Authors:** Janghan Choi, Emma Traylor, Rachel Husak, Annabelle Foster, Aubrey Akere-Nkongho Tambe

**Affiliations:** Department of Animal and Food Sciences, Texas Tech University, Lubbock, TX 79409, USA; emtraylo@ttu.edu (E.T.); rhusak@ttu.edu (R.H.); annabfos@ttu.edu (A.F.); autambe@ttu.edu (A.A.-N.T.)

**Keywords:** IUGR piglets, swine production, omics, molecular analysis, gut health, oxidative stress, and meat quality

## Abstract

Intrauterine growth retardation (IUGR) is highly prevalent in modern swine production, and many affected piglets survive past weaning and are raised for commercial pork production. This review summarizes the current understanding of the physiological challenges of IUGR piglets from a molecular perspective and evaluates recent advances in nutritional strategies aimed at mitigating their negative outcomes. Molecular approaches, including omics technologies and targeted analyses, have been employed to investigate the physiological characteristics of IUGR piglets. These approaches consistently show that IUGR piglets exhibit systemic dysfunction, including compromised gut health, increased inflammation and oxidative stress, and impaired function of multiple organs such as the intestine, liver, kidney, and immune-related tissues. Moreover, IUGR piglets often display poor muscle development and meat quality. The multifactorial nature of these issues suggests that targeting a single physiological parameter may be insufficient, and comprehensive interventions are needed to address the widespread effects of IUGR. Promising nutritional strategies such as supplementation with polyphenol-rich plant extracts, amino acids, and probiotics have demonstrated potential in improving gut integrity, beneficially modulating microbiota, and enhancing the overall health and performance of IUGR piglets. By supporting the systemic recovery of IUGR piglets, nutritional interventions could improve overall productivity in swine production systems.

## Simple Summary

Intrauterine growth retardation (IUGR) is a common issue in modern swine production, leading to the birth of piglets that are smaller and developmentally delayed. Although many IUGR piglets survive past weaning and eventually reach market weight, they often experience suboptimal growth and face long-term health challenges. Studies using advanced molecular tools have shown that IUGR piglets suffer from compromised gut health, increased inflammation and oxidative stress, and systemic organ dysfunction, including the intestine, liver, kidney, and immune system. These piglets also exhibit poor muscle growth and reduced meat quality, indicating systemic problems rather than isolated defects. Recent research suggests that nutritional interventions including plant extracts rich in polyphenols, amino acids, and probiotics can enhance growth, gut function, and overall health in IUGR piglets. Addressing these challenges through comprehensive nutritional strategies may help improve growth performance, animal health, and production efficiency in swine production.

## 1. Introduction

Advances in genetic, nutritional, and management aspects of sow production increases litter size, which can increase profitability for swine producers [1]. The average litter size per sow has increased substantially in recent decades, from approximately 4 to 5 piglets per litter between 1950 and 2000 [2], to 11.7 piglets in 2000 [3], and further to 17.2 piglets by 2018 in Denmark [4]. While increasing the number of piglets born and improving overall productivity, they are often associated with negative outcomes for piglet health. These include compromised immune and digestive function, reduced birth weight, and lower survival rates through weaning [5,6]. This is primarily caused by an increased number of fetuses within a limited uterine space, leading to reduced piglet birth weight.

**Figure 1:**
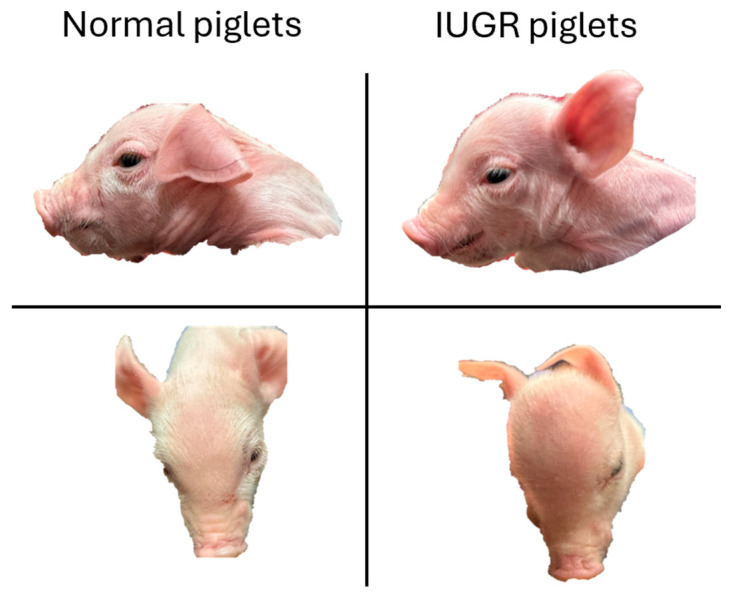
Characteristics of intrauterine growth retarded (IUGR) piglets: One of the most recognizable features of IUGR is their distinct head shape, often described as a “dolphin-like” profile. Pictures were taken in New Deal Swine facility at Texas Tech University, Lubbock, Texas.

Intrauterine growth restriction (IUGR), a condition characterized by impaired fetal growth within the womb, poses a significant challenge in hyperprolific sows commonly found in modern swine production systems [7]. The incidence rate of IUGR piglets is approximately 5 to 10% in newborn piglets [8]. In Denmark, which is known to have the highest litter size, the incidence of IUGR could be estimated to be up to 30% [9]. As shown in Figure 1, one of the most recognizable features of IUGR piglets is their distinct head shape, often described as a “dolphin-like” profile, as noted by Bahnsen et al. [10]. This phenomenon occurs when placental insufficiency, often exacerbated by spatial limitations in the uterus, leads to a restricted supply of nutrients and oxygen during gestation [11]. In response, the fetus redistributes blood flow preferentially towards vital organs, particularly the brain, heart, and adrenal glands to support their development at the expense of other tissues. The IUGR piglets exhibit reduced growth performance and compromised functionality of organs compared to normal piglets, which may negatively impact production efficiency and meat quality [12]. The IUGR piglets have higher preweaning mortality rates compared to normal piglets [12]. However, with proper management during the nursery phase, many of these piglets can survive, sustain growth, and ultimately reach market weight [13]. To better understand and address the long-term consequences of IUGR, it is essential to investigate the underlying biological mechanisms driving these outcomes.

Molecular-level investigations using omics technologies and targeted analyses can provide valuable insights into the biological mechanisms that contribute to impaired growth and health outcomes of IUGR piglets. Omics involves the comprehensive analysis and quantification of entire sets of biological molecules, examining their contribution to the structure, function, and dynamics of an organism or a group of organisms [14]. Entire sets of DNA, RNA, proteins, metabolites can be investigated by utilizing genomics, transcriptomics, proteomics, and metabolomics. These omics techniques can provide valuable insights for identifying the precise etiologies of diseases and for developing effective intervention strategies [15,16,17]. These approaches also help identify nutritional strategies that may reduce the severity of IUGR. This review aims to provide an overview of the physiological challenges faced by IUGR piglets through a molecular perspective with omics approach and to assess recent progress in nutritional strategies designed to alleviate their adverse effects.

## 2. Gut Development and Gut Barrier Integrity

Proper gut development during early life is essential for pigs to achieve optimal nutrient digestion and absorption. This development can be assessed through the analysis of intestinal morphology and expression of genes related to gut development and gut barrier integrity. Hu et al. [18] and Li et al. [19] showed that the IUGR piglets had compromised gut structure, and the supplementation of *Bacillus subtilis* induced gut development by increasing abundance of tight junction proteins in the small intestine of IUGR piglets. However, Santos et al. [20] showed that IUGR piglets had compromised duodenal structure after 70 days of age. Furthermore, Wang et al. [21] demonstrated that expression of genes related to gut development were altered in IUGR piglets. The compromised gut development was accompanied by decreased digestive enzyme activities and nutrient utilization [20,22,23]. Moreover, diverse nutrient transporters in the small intestine were significantly downregulated in IUGR piglets potentially due to poor gut development [24]. The compromised intestinal development in IUGR piglets, characterized by structural abnormalities, altered gene expression, and reduced digestive and absorptive capacity, demonstrated the importance of early nutritional interventions to support gut maturation and improve long-term growth performance.

Gut barrier integrity, acting as a selective barrier that allows nutrient absorption while preventing harmful pathogens, toxins, and antigens from entering the bloodstream, is a key immune trait that helps reduce systemic infections and inflammation in animals [25]. The reduced expression of tight junction proteins in IUGR piglets indicates impaired gut barrier function, which may contribute to increased intestinal permeability and reduced health status [26,27,28,29,30]. Table 1 shows various nutritional interventions that improved the gut barrier integrity of IUGR piglets. Enhanced gut barrier integrity is often linked to improved gut structure, potentially due to reduced inflammation and increased cellular regeneration in the intestinal lining [31]. This structural improvement is commonly accompanied by enhanced gut functionality. A previous study by Chen et al. [32] demonstrated that upregulated tight junction proteins resulted in enhanced gut barrier integrity in IUGR piglets. Nevertheless, most studies have focused on the gene expression of tight junction proteins, increased expression alone does not necessarily translate to improved gut barrier integrity because mRNA levels do not guarantee corresponding protein translation, proper localization, or functional assembly of tight junctions [33]. Therefore, more in vivo or ex vivo permeability assessments are needed in IUGR piglets to insightfully evaluate gut barrier function.

## 3. Microbiota and Metabolites in Gut Digesta

A compromised gut ecosystem, defined by altered gut microbiota, impaired intestinal structure, and disrupted microbial metabolite production along with poor overall animal health, significantly contributes to reduced growth performance in IUGR piglets [38]. The 16S rRNA analysis refers to an omics approach used to investigate entire bacterial populations by sequencing the 16S rRNA genes, which are universally present in all bacteria. Greater alpha diversity indices suggest greater richness and evenness, which is associated with the maturity of the gut ecosystem [39]. Beta diversity reflects the differences in microbial community composition between samples, providing insights into the structural variation and heterogeneity of the gut microbiota among individuals or groups [40]. The phylum Firmicutes and Bacteroidetes are the dominant group in pig microbiota and play important roles in producing short chain fatty acids (SCFA) [41], which are important energy sources for the host [42,43]. The higher abundance of the phylum Firmicutes and Bacteroidetes and the greater ratio of Firmicutes to Bacteroidetes indicates a mature gut ecosystem with greater production of SCFA. Proteobacteria includes diverse pathogenic bacterial groups such as *E.coli*, *Salmonella* spp. Helicobacter, etc., [44]. As shown in Table 2, IUGR piglets exhibited compromised gut microbiota, characterized by a reduced abundance of Firmicutes and Bacteroidetes, as well as a lower Firmicutes-to-Bacteroidetes ratio, alongside an increased presence of pathogenic bacteria in various ages. Moreover, the metabolic profile of the digesta was adversely affected, accompanied by disruptions in the gut microbiota composition of IUGR piglets [45,46]. A previous study by Cui et al. [24] demonstrated that IUGR piglets with catch-up growth by weaning age exhibited enhanced alpha diversity along with an increased abundance of beneficial bacteria and a reduced prevalence of pathogenic bacteria compared to IUGR piglets without catch-up growth. However, Che et al. [47] and Che et al. [35] reported that IUGR piglets in the mid-nursery phase had microbial populations similar to those of normal piglets, suggesting that IUGR piglets may catch up in microbiota development. Cui et al. [24] suggested that some IUGR piglets may be able to catch up to their normal piglets due to having a lower abundance of pathogenic microbiota. Thus, modulating gut microbiota may help mitigate the negative effects of IUGR in piglets.

Various nutritional interventions including diverse probiotics and plant extracts were evaluated to enhance the gut microbiota of IUGR piglets as shown in Table 3. While some studies have shown that improving gut microbiota enhanced growth performance [19,26,51], others have reported no such effect [35,36,52]. Improvement of the gut microbiota does not always lead to enhanced growth performance, but it may offer protection against challenges such as microbial infections or heat stress [53,54]. Most studies investigating the effects of nutritional interventions on the gut microbiota of pigs with IUGR have focused on the suckling and weaning phases. Nevertheless, a previous study by Xiong et al. [50] reported that IUGR piglets in the grower–finisher phase (25 to 100 kg) exhibited compromised gut microbiota. These findings underscore the need for further research to explore the long-term effects of nutritional interventions in IUGR during later growth stages.

## 4. Local and Systemic Inflammation

Inflammation is essential for combating pathogens and initiating tissue repair [55,56,57]. However, both acute and chronic inflammation can negatively affect the health and productivity of pigs [58]. It has been demonstrated that piglets exhibiting IUGR traits are characterized by chronic low-grade systemic inflammation, often evidenced by persistently elevated levels of pro-inflammatory cytokines [59]. It may originate from compromised gut ecosystems [60] and/or dysfunction of organs such as liver and immune organs including the thymus, spleen, and lymphoid tissues [61,62]. Furthermore, the compromised gut microbiota with more abundance of pathogenic bacteria as shown in Table 2 may induce more systemic infection and inflammation in piglets with IUGR traits. Various studies have demonstrated that IUGR piglets exhibit upregulated gene and protein expression of pro-inflammatory cytokines including toll-like receptor 4 (TLR4), interleukin-1 beta (IL-1β), nuclear factor kappa B (NF-κB), tumor necrosis factor-alpha (TNF-α), and interleukin-6 (IL-6) in both the liver and intestine [19,63,64,65]. A previous study by Dong et al. [61] suggested that intestinal inflammation in IUGR piglets may result from the underdevelopment of key immune organs such as the thymus, spleen, and mesenteric lymph nodes which can impair immune regulation in the gut. Intrauterine malnutrition may also induce autophagosome activity, indicating cellular stress and further contributing to inflammation. Moreover, diverse studies demonstrated that IUGR piglets exhibited inflammations in the liver [59,64], kidney [66], and brain [67]. Amdi et al. [68] reported that IUGR piglets exhibited lower levels of CD4^+^ T cells and IL-1β in the blood, suggesting an altered immune response compared to normal piglets. Furthermore, Huang et al. [49] demonstrated that IUGR piglets had different systemic inflammatory profile compared to the normal piglets. Inflammation appears to be widespread in IUGR piglets, affecting multiple organs. Supplementation with plant extracts rich in polyphenolic compounds has been shown to alleviate both intestinal and systemic inflammation in IUGR piglets [63,65]. While nutrient-dense milk replacer alleviated impairments in systemic and gut immune function [69], high-fat diets exacerbated hepatic inflammation in IUGR piglets [59]. These findings highlight the importance of targeted nutritional interventions to mitigate inflammation and improve health outcomes in IUGR piglets.

## 5. Oxidative Stress

Oxidative stress typically refers to an imbalance between oxidants and antioxidants within cells, leading to oxidative damage of cellular macromolecules, cell death through apoptosis or necrosis, and structural damage to tissues [56,70,71]. Oxidative stress is closely associated with inflammation since the causing factors are known to xenobiotics, pathogens, and stimulated inflammation [72]. The antioxidant defense system can be modulated by enzymatic antioxidants such as superoxide dismutase (SOD), catalase (CAT), and glutathione peroxidase (GPx), as well as non-enzymatic antioxidants like glutathione, vitamin E, and vitamin C. A recent study by Gao et al. [73] utilized metabolomics and transcriptomics to demonstrate that IUGR exhibited various metabolic abnormalities such as mitochondrial dysfunction, imbalanced fatty acid composition, disrupted sources of one-carbon unit supply, and impaired galactose conversion, which may contribute to hepatic oxidative stress. Moreover, studies demonstrated that hepatic oxidative stress in IUGR piglets [64,74]. Liver mitochondria in IUGR piglets exhibited impaired function characterized by excessive swelling, overproduction of superoxide radicals, and elevated malondialdehyde levels, indicating increased oxidative stress. This heightened oxidative stress may lead to a stronger activation of the antioxidant defense system compared to normal piglets [75]. Various studies have reported that oxidative stress is induced in the intestines of IUGR piglets, as evidenced by colorimetric assays, ELISA, and gene expression analyses [27,29,76]. Bioactive compounds such as plant extracts [76,77,78,79], bile acids [80], epidermal growth factor [81], and methionine [76] improved antioxidant status of different organs such as systemic circulation, gut, and liver in IUGR piglets. Hence, IUGR piglets experienced systemic oxidative stress, which may be alleviated by supplementing antioxidant-related compounds including plant extracts and methionine.

## 6. Muscle Development and Meat Quality

IUGR piglets, which represent about 5–10% of the population, exhibit poor growth, higher preweaning mortality, and altered muscle development, making it important to understand their muscle and meat quality to improve production outcomes and meat value [13]. Hu et al. [82] reported that IUGR impaired skeletal muscle growth and disrupted hormonal and gene expression related to energy metabolism, leading to greater energy deficits under postnatal nutritional restriction especially in the nursery phase. This restriction further delayed myofiber maturation, potentially due to shifts in myosin heavy chain isoform expression and metabolic status. Some studies have reported no significant differences in slaughter body weight between IUGR and normal pigs from D 110 to 200 [79,83], while others have observed significant differences [13,79]. These results suggest that the lack of significant differences in meat yield between IUGR and normal piglets may be due to compensatory and accelerated growth during the finishing phase, leading to a convergence in slaughter body weight. However, the absence of differences in carcass weight does not necessarily imply similar tissue composition. Therefore, it is important to further examine whether the increased body mass is primarily attributed to fat accumulation rather than lean tissue growth. This rapid catch-up growth could be a contributing factor affecting meat quality [84]. Zhang et al. [79] demonstrated that IUGR piglets had increased oxidative stress and reduced antioxidant enzyme activities in leg muscles compared to normal piglets on the day of slaughter (D 115). Li et al. [83] demonstrated that IUGR piglets exhibited increased oxidative stress, which was associated with greater fat deposition, reduced pH, and compromised meat color in the longissimus dorsi muscle at D 200. Zhang et al. [13] showed that the longissimus dorsi muscle exhibited a shift toward less favorable muscle fiber types, along with reduced water holding capacity and inferior meat color in slaughter pigs. Nevertheless, Matyba et al. [85] reported that whereas IUGR piglets had compromised meat quality, characterized by lower pH, higher electrical conductivity, and lower water holding capacity, their meat was more tender and received higher sensory scores for taste, aroma, and overall acceptability. Although inconsistencies persist in parameters such as slaughter weight, meat color, and pH, pork from IUGR pigs is generally marked by increased fat content, reduced water-holding capacity, and heightened oxidative stress. Hu et al. [86] demonstrated that high nutrient intake during the suckling period enhances skeletal muscle growth and maturity in IUGR piglets by upregulating genes related to protein deposition and promoting the development of glycolytic-type myofibers. However, a previous study by Liu et al. [87] reported that high-fat diets exacerbated metabolic dysfunction in IUGR piglets by impairing mitochondrial respiration, reducing mtDNA and energy-related metabolites, and downregulating genes involved in mitochondrial biogenesis and function in skeletal muscle. Numerous studies have examined the impact of nutritional interventions on oxidative status and meat quality in IUGR piglets (Table 4). Therefore, the meat from IUGR piglets is characterized by a low water-holding capacity, impaired oxidative stability, poor meat color, and increased fat content. Supplementation with bioactive compounds such as choline, glycine, and plant extract rich in polyphenols has been shown to improve these meat quality parameters in IUGR piglets.

## 7. Conclusions

**Figure 2:**
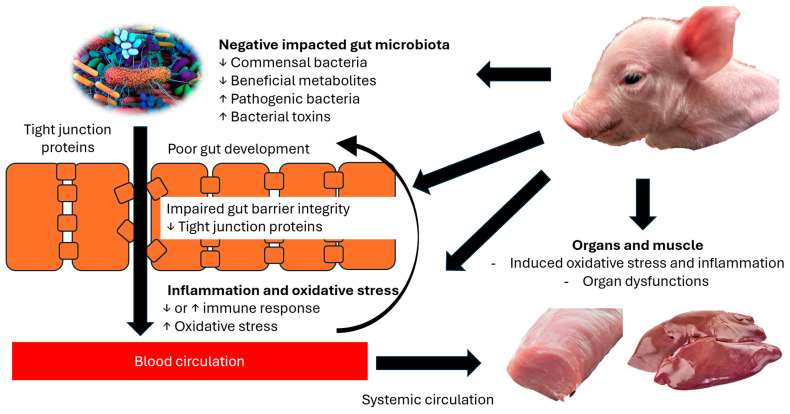
Interconnected and direct effects of IUGR on gut and systemic health in piglets. IUGR can directly impair multiple aspects of gut and systemic health including gut microbiota, intestinal barrier integrity, inflammation, oxidative stress, and organ function. These parameters are also closely interconnected, amplifying the overall impact. IUGR alters the gut microbiota by decreasing commensal bacteria and beneficial metabolites while increasing pathogenic bacteria and bacterial toxins. These microbial changes weaken gut barrier function by reducing tight junction protein expression, leading to increased intestinal permeability. The subsequent translocation of harmful microbial products contributes to immune dysregulation and elevated oxidative stress, which, in turn, impairs the function of vital organs and skeletal muscle.

Omics and molecular-based approaches offer valuable insights into the complex biological mechanisms underlying IUGR, enabling the identification of key pathways and potential targets for intervention. IUGR piglets commonly exhibit disrupted gut microbiota, compromised intestinal barrier function, heightened inflammation and oxidative stress, impaired muscle development, and reduced meat quality. These interconnected issues not only directly affect piglet health and growth but also reflect broader systemic dysfunctions (Figure 2). While addressing individual traits may provide some benefit, a more integrated strategy that considers these multifactorial challenges is likely to be more effective. Targeted nutritional interventions designed to support the recovery and development of IUGR piglets hold promise for improving their overall health and productivity, ultimately enhancing the efficiency and sustainability of modern swine production systems.

## Figures and Tables

**Table 1:** Summary of effects of nutritional interventions on gut barrier integrity changes in intrauterine growth retarded (IUGR) pigs in the weaning phase.

References	Nutritional Interventions	Analysis and Conditions	Observations in the Small Intestine
[26]	Dihydroartemisinin	Gene and protein expression	Upregulated claudin-1 and occludin
[34]	Epidermal growth factor	Gene expression	Upregulated zonula occludens-1, claudin-1, occludin, and mucin 2
[35]	Flaxseed oil	Gene expression	Upregulated claudin-1 and zonula occludens-1
[36]	*Bacillus subtilis*	Gene expression	Upregulated zonula occludens-1, occludin, and claudin-1
[37]	Equol	Gene expression	Upregulated zonula occludens-1, claudin-1, occluding, mucin-2, and trefoil factor-3
[32]	Resveratrol and its derivative pterostilbene	Gene and protein expression and metabolites	Decreased plasma D-lactate concentration and upregulated occludens-1 and zonula occludens-1
[31]	*Lactobacillus amylovorus*	Gene expression and metabolites	Decreased plasma D-lactate concentration and upregulated claudin-1 and zonula occludens-1

**Table 2:** Summary of changes in microbiota and metabolites in gut content of intrauterine growth retarded (IUGR) pigs at different stages.

References	Analysis and Conditions	Observations
[48]	Jejunal and ileal content microbiota on D 7, 21 (weaning), and 28	Decreased alpha diversity Decreased the abundance of Firmicutes and Bacteroidetes Increased the abundance of Proteobacteria, Pasteurella, and *Escherichia*-shigella
[46]	Colonic content microbiota on 7, 21 (weaning), and 28 days of age	Decreased alpha diversity Decreased the abundance of Firmicutes and Bacteroidetes
[46]	Colonic content metabolomics on 7, 21, and 28 days of age	Affected amino sugar, nucleotide sugar, and aromatic amino acid metabolism.
[49]	Fecal microbiota at birth and 12 h	Increased the abundance of Proteobacteria and Escherichia-shigella Decreased the abundance of Firmicutes
[47]	Colonic content microbiota at D 28 and 35 after weaning at D 21	Did not significantly affect microbiota (alpha diversity and taxa abundance)
[50]	Jejunal content microbiota at 25, 50, and 100 kg body weight	Increased the abundance of Firmicutes, Ruminococcaceae, and Lactobacillus
[45]	Colonic content microbiota and at 25, 50, and 100 kg body weight	Decreased the abundance of Firmicutes Decreased the ratio of Firmicutes/Bacteroidetes
[45]	Colonic content metabolomics at 25, 50, and 100 kg body weight	Decreased short chain fatty acid production Increased colonic bioamines Disrupted colonic barrier function and induced inflammation Suppressed lipid metabolism

**Table 3:** Summary of effects of nutritional interventions on gut microbiota and metabolomic changes in intrauterine growth retarded (IUGR) pigs at different stages.

References	Nutritional Interventions	Sample	Phase	Observations
[18]	*Bacillus subtilis* PB6	Colon digesta	Suckling	Did not significantly influence gut microbiota
[51]	*Clostridium butyricum*	Ileum digesta	Suckling	Decreased the abundance of Streptococcus and Enterococcus
[36]	*Bacillus subtilis*	Jejunum digesta	Suckling	Decreased the abundance of Bacteroidetes and Proteobacteria
[52]	Bile acid	Colon digesta	Weaning	Increased the abundance of Firmicutes and Bacteroidetes abundance
[19]	*Bacillus amyloliquefaciens*	Jejunum digesta Ileal digesta	Weaning	Decreased the abundance of *E. coli* Increased the abundance of Lactobacillus and Bifidobacterium
[35]	Flaxseed oil	Colon digesta	Weaning	Decreased the abundance of pathogenic bacteria including *Spirochaetes*, and increased *Actinobacteria*, and *Blautia* and *Bifidobacterium* in colonic digesta.
[32]	Resveratrol and its derivative pterostilbene	Cecum digesta	Weaning	Increased the abundance of Bacteroidetes, Faecalibacterium, and Prevotella, and decreased the abundance of Proteobacteria and *Escherichia coli*
[26]	Dihydroartemisinin	Jejunum digesta	Weaning	Improved alpha diversity Increased the abundance of Actinobacteria, *Streptococcus*, *Blautia*, and *Streptococcus*

**Table 4:** Summary of effects of nutritional interventions on meat quality of intrauterine growth retarded (IUGR) pigs at different body weights and ages.

References	Bioactive Compounds	Age	Body Weight	Meat Part	Observations
[79]	Curcumin	D 115	53 to 57 kg	Leg meat	Reduced malondialdehyde levels Enhanced antioxidant capacity by upregulating catalase, superoxide dismutase, and peroxidase Improved meat quality by decreasing drip loss and enhancing meat color
[88]	Resveratol	D 150		*Longissimus lumborum*	Increased glutathione peroxidase activity and Myosin Heavy Chain 1 gene expression Reduced malondialdehyde levels Enhanced fatty acid oxidation via upregulated PPARα and targeted genes expression Improved meat quality by decreasing drip loss and enhancing meat color
[83]	Choline	D 200	100 to 114 kg	Longissimus dorsi	Increased malondialdehyde levels and increased oxidative stress Reduced the fat deposition
[89]	Glycine	D 188	118 to 134 kg	Longissimus thoracis	Enhanced meat color Reducing backfat thickness

## Data Availability

Not applicable.

## References

[1] Gormley A., Jang K.B., Garavito-Duarte Y., Deng Z., Kim S.W. (2024). Impacts of maternal nutrition on sow performance and potential positive effects on piglet performance. Animals.

[2] Knap P.W., Knol E.F., Sørensen A.C., Huisman A.E., van der Spek D., Zak L.J., Granados Chapatte A., Lewis C.R. (2023). Genetic and phenotypic time trends of litter size, piglet mortality, and birth weight in pigs. Front. Anim. Sci..

[3] Rutherford K., Baxter E.M., Ask B., Berg P., D’Eath R.B., Jarvis S., Jensen K.K., Lawrence A., Moustsen V., Robson S. (2011). The Ethical and Welfare Implications of Large Litter Size in the Domestic Pig: Challenges and Solutions.

[4] SEGES (2019). Nøgetal for Økologisk Sohold. https://www.landbrugsinfo.dk/-/media/landbrugsinfo/basic/5/9/0/oe-noegletal-gns-oekologoisk_sohold-2018.pdf.

[5] Zindove T.J., Mutibvu T., Shoniwa A.C., Takaendesa E.L. (2021). Relationships between litter size, sex ratio and within-litter birth weight variation in a sow herd and consequences on weaning performance. Transl. Anim. Sci..

[6] Zhu H., Liu Y., Xie X., Huang J., Hou Y. (2013). Effect of L-arginine on intestinal mucosal immune barrier function in weaned pigs after Escherichia coli LPS challenge. Innate Immun..

[7] Wang J., Feng C., Liu T., Shi M., Wu G., Bazer F.W. (2017). Physiological alterations associated with intrauterine growth restriction in fetal pigs: Causes and insights for nutritional optimization. Mol. Reprod. Dev..

[8] Pieszka M., Szczurek P., Orczewska-Dudek S., Kamyczek M., Pieszka M. (2023). Determining the effect of pancreatic-like enzymes (PLEMs) added to the feed of pregnant sows on fetal size of piglets to minimize IUGR syndrome caused by fetal malnutrition. Animals.

[9] Riddersholm K.V., Bahnsen I., Bruun T.S., de Knegt L.V., Amdi C. (2021). Identifying risk factors for low piglet birth weight, high within-litter variation and occurrence of intrauterine growth-restricted piglets in hyperprolific sows. Animals.

[10] Bahnsen I., Riddersholm K.V., de Knegt L.V., Bruun T.S., Amdi C. (2021). The effect of different feeding systems on salivary cortisol levels during gestation in sows on herd level. Animals.

[11] Hansen C., Hales J., Amdi C., Moustsen V. (2018). Intrauterine growth-restricted piglets defined by their head shape have impaired survival and growth during the suckling period. Anim. Prod. Sci..

[12] Van Ginneken C., Ayuso M., Van Bockstal L., Van Cruchten S. (2023). Preweaning performance in intrauterine growth-restricted piglets: Characteristics and interventions. Mol. Reprod. Dev..

[13] Zhang L., Wang Y., Kong Y., Ahmad H., Yan R., Dong L., Zhang J., Wang T. (2018). Effects of intrauterine growth retardation on growth, meat quality and muscle fiber composition of pigs. Pak. J. Zool..

[14] Yamada R., Okada D., Wang J., Basak T., Koyama S. (2021). Interpretation of omics data analyses. J. Hum. Genet.

[15] Kong B., Owens C., Bottje W., Shakeri M., Choi J., Zhuang H., Bowker B. (2024). Proteomic analyses on chicken breast meat with white striping myopathy. Poult. Sci..

[16] Choi J., Shakeri M., Kim W.K., Kong B., Bowker B., Zhuang H. (2024). Comparative metabolomic analysis of spaghetti meat and wooden breast in broiler chickens: Unveiling similarities and dissimilarities. Front. Physiol..

[17] Zhou H., Quach A., Nair M., Abasht B., Kong B., Bowker B. (2025). Omics based technology application in poultry meat research. Poult. Sci..

[18] Hu L., Peng X., Chen H., Yan C., Liu Y., Xu Q., Fang Z., Lin Y., Xu S., Feng B. (2017). Effects of intrauterine growth retardation and Bacillus subtilis PB6 supplementation on growth performance, intestinal development and immune function of piglets during the suckling period. Eur. J. Nutr..

[19] Li Y., Zhang H., Su W., Ying Z., Chen Y., Zhang L., Lu Z., Wang T. (2018). Effects of dietary Bacillus amyloliquefaciens supplementation on growth performance, intestinal morphology, inflammatory response, and microbiota of intra-uterine growth retarded weanling piglets. J. Anim. Sci. Biotechnol..

[20] Santos T.G., Fernandes S.D., de Oliveira Araújo S.B., Felicioni F., de Mérici Domingues e Paula T., Caldeira-Brant A.L., Ferreira S.V., de Paula Naves L., de Souza S.P., Campos P.H.R.F. (2022). Intrauterine growth restriction and its impact on intestinal morphophysiology throughout postnatal development in pigs. Sci. Rep..

[21] Wang T., Huo Y.J., Shi F., Xu R.J., Hutz R.J. (2005). Effects of intrauterine growth retardation on development of the gastrointestinal tract in neonatal pigs. Neonatology.

[22] Ferenc K., Pietrzak P., Godlewski M.M., Piwowarski J., Kilianczyk R., Guilloteau P., Zabielski R. (2014). Intrauterine growth retarded piglet as a model for humans–studies on the perinatal development of the gut structure and function. Reprod. Biol..

[23] Li T., Huang S., Lei L., Tao S., Xiong Y., Wu G., Hu J., Yuan X., Zhao S., Zuo B. (2021). Intrauterine growth restriction alters nutrient metabolism in the intestine of porcine offspring. J. Anim. Sci. Biotechnol..

[24] Cui C., Wu C., Wang J., Ma Z., Zheng X., Zhu P., Wang N., Zhu Y., Guan W., Chen F. (2022). Restored intestinal integrity, nutrients transporters, energy metabolism, antioxidative capacity and decreased harmful microbiota were associated with IUGR piglet’s catch-up growth before weanling. J. Anim. Sci. Biotechnol..

[25] Choi J., Wang L., Liu S., Lu P., Zhao X., Liu H., Lahaye L., Santin E., Liu S., Nyachoti M. (2020). Effects of a microencapsulated formula of organic acids and essential oils on nutrient absorption, immunity, gut barrier function, and abundance of enterotoxigenic Escherichia coli F4 in weaned piglets challenged with *E. coli* F4. J. Anim. Sci..

[26] Niu Y., Zhang R., Yang C., He J., Wang T. (2024). Dietary supplementation with dihydroartemisinin improves intestinal barrier function in weaned piglets with intrauterine growth retardation by modulating the gut microbiota. J. Anim. Sci..

[27] Tang X., Xiong K. (2022). Intrauterine growth retardation affects intestinal health of suckling piglets via altering intestinal antioxidant capacity, glucose uptake, tight junction, and immune responses. Oxid. Med. Cell. Longev..

[28] Qi M., Tan B., Wang J., Liao S., Li J., Cui Z., Shao Y., Ji P., Yin Y. (2021). Postnatal growth retardation is associated with deteriorated intestinal mucosal barrier function using a porcine model. J. Cell. Physiol..

[29] Wang W., Degroote J., Van Ginneken C., Van Poucke M., Vergauwen H., Dam T.M.T., Vanrompay D., Peelman L.J., De Smet S., Michiels J. (2016). Intrauterine growth restriction in neonatal piglets affects small intestinal mucosal permeability and mRNA expression of redox-sensitive genes. FASEB J..

[30] Huang S., Wu Z., Yuan X., Li N., Li T., Wang J., Levesque C.L., Feng C. (2020). Transcriptome differences suggest novel mechanisms for intrauterine growth restriction mediated dysfunction in small intestine of neonatal piglets. Front. Physiol..

[31] Wu Y., Liu X., Zou Y., Zhang X., Wang Z., Hu J., Han D., Zhao J., Dai Z., Wang J. (2024). Lactobacillus amylovorus promotes lactose utilization in small intestine and enhances intestinal barrier function in intrauterine growth restricted piglets. J. Nutr..

[32] Chen Y., Zhang H., Chen Y., Jia P., Ji S., Zhang Y., Wang T. (2021). Resveratrol and its derivative pterostilbene ameliorate intestine injury in intrauterine growth-retarded weanling piglets by modulating redox status and gut microbiota. J. Anim. Sci. Biotechnol..

[33] Camilleri M. (2019). Leaky gut: Mechanisms, measurement and clinical implications in humans. Gut.

[34] Tang X., Xiong K. (2022). Dietary epidermal growth factor supplementation alleviates intestinal injury in piglets with intrauterine growth retardation via reducing oxidative stress and enhancing intestinal glucose transport and barrier function. Animals.

[35] Che L., Zhou Q., Liu Y., Hu L., Peng X., Wu C., Zhang R., Tang J., Wu F., Fang Z. (2019). Flaxseed oil supplementation improves intestinal function and immunity, associated with altered intestinal microbiome and fatty acid profile in pigs with intrauterine growth retardation. Food Funct..

[36] Yun Y., Ji S., Yu G., Jia P., Niu Y., Zhang H., Zhang X., Wang T., Zhang L. (2021). Effects of Bacillus subtilis on jejunal integrity, redox status, and microbial composition of intrauterine growth restriction suckling piglets. J. Anim. Sci..

[37] Zhang Y., Ren J., Chen L., Yan H., Zou T., Zhang H., Liu J. (2023). Effects of equol supplementation on growth performance, redox status, intestinal health and skeletal muscle development of weanling piglets with intrauterine growth retardation. Animals.

[38] D’Inca R., Kloareg M., Gras-Le Guen C., Le Huërou-Luron I. (2010). Intrauterine growth restriction modifies the developmental pattern of intestinal structure, transcriptomic profile, and bacterial colonization in neonatal pigs. J. Nutr..

[39] Massacci F.R., Berri M., Lemonnier G., Guettier E., Blanc F., Jardet D., Rossignol M.N., Mercat M.-J., Doré J., Lepage P. (2020). Late weaning is associated with increased microbial diversity and Faecalibacterium prausnitzii abundance in the fecal microbiota of piglets. Anim. Microbiome.

[40] Kers J.G., Saccenti E. (2022). The power of microbiome studies: Some considerations on which alpha and beta metrics to use and how to report results. Front. Microbiol..

[41] Liu P., Zhao J., Guo P., Lu W., Geng Z., Levesque C.L., Johnston L.J., Wang C., Liu L., Zhang J. (2017). Dietary corn bran fermented by Bacillus subtilis MA139 decreased gut cellulolytic bacteria and microbiota diversity in finishing pigs. Front. Cell. Infect. Mi..

[42] Che D., Adams S., Wei C., Gui-Xin Q., Atiba E.M., Hailong J. (2019). Effects of Astragalus membranaceus fiber on growth performance, nutrient digestibility, microbial composition, VFA production, gut pH, and immunity of weaned pigs. Microbiologyopen.

[43] Choi J., Ko H., Tompkins Y.H., Teng P.-Y., Lourenco J.M., Callaway T.R., Kim W.K. (2021). Effects of Eimeria tenella infection on key parameters for feed efficiency in broiler chickens. Animals.

[44] Ma X., Wang Q., Li H., Xu C., Cui N., Zhao X. (2017). 16S rRNA genes Illumina sequencing revealed differential cecal microbiome in specific pathogen free chickens infected with different subgroup of avian leukosis viruses. Vet. Microbiol..

[45] Xiong L., Azad M.A.K., Liu Y., Zhang W., Zhu Q., Hu C., You J., Kong X. (2024). Intrauterine Growth Restriction Affects Colonic Barrier Function via Regulating the Nrf2/Keap1 and TLR4-NF-κB/ERK Pathways and Altering Colonic Microbiome and Metabolome Homeostasis in Growing–Finishing Pigs. Antioxidants.

[46] Tang W., Zhang W., Azad M.A.K., Ma C., Zhu Q., Kong X. (2022). Metabolome, microbiome, and gene expression alterations in the colon of newborn piglets with intrauterine growth restriction. Front. Microbiol..

[47] Che L., Hu L., Zhou Q., Peng X., Liu Y., Luo Y., Fang Z., Lin Y., Xu S., Feng B. (2020). Microbial insight into dietary protein source affects intestinal function of pigs with intrauterine growth retardation. Eur. J. Nutr..

[48] Zhang W., Ma C., Xie P., Zhu Q., Wang X., Yin Y., Kong X. (2019). Gut microbiota of newborn piglets with intrauterine growth restriction have lower diversity and different taxonomic abundances. J. Appl. Microbiol..

[49] Huang S., Li N., Liu C., Li T., Wang W., Jiang L., Li Z., Han D., Tao S., Wang J. (2019). Characteristics of the gut microbiota colonization, inflammatory profile, and plasma metabolome in intrauterine growth restricted piglets during the first 12 hours after birth. J. Microbiol..

[50] Xiong L., You J., Zhang W., Zhu Q., Blachier F., Yin Y., Kong X. (2020). Intrauterine growth restriction alters growth performance, plasma hormones, and small intestinal microbial communities in growing-finishing pigs. J. Anim. Sci. Biotechnol..

[51] Zhang X., Yun Y., Lai Z., Ji S., Yu G., Xie Z., Zhang H., Zhong X., Wang T., Zhang L. (2023). Supplemental Clostridium butyricum modulates lipid metabolism by reshaping the gut microbiota composition and bile acid profile in IUGR suckling piglets. J. Anim. Sci. Biotechnol..

[52] Liu Y., Azad M.A.K., Ding S., Zhu Q., Blachier F., Yu Z., Gao H., Kong X. (2023). Dietary bile acid supplementation in weaned piglets with intrauterine growth retardation improves colonic microbiota, metabolic activity, and epithelial function. J. Anim. Sci. Biotechnol..

[53] Xia B., Wu W., Fang W., Wen X., Xie J., Zhang H. (2022). Heat stress-induced mucosal barrier dysfunction is potentially associated with gut microbiota dysbiosis in pigs. Anim. Nutr..

[54] Fouhse J., Zijlstra R., Willing B. (2016). The role of gut microbiota in the health and disease of pigs. Anim. Front..

[55] Eming S.A., Wynn T.A., Martin P. (2017). Inflammation and metabolism in tissue repair and regeneration. Science.

[56] Lauridsen C. (2019). From oxidative stress to inflammation: Redox balance and immune system. Poult. Sci..

[57] Mogensen T.H. (2009). Pathogen recognition and inflammatory signaling in innate immune defenses. Clin. Microbiol. Rev..

[58] Liu Y. (2015). Fatty acids, inflammation and intestinal health in pigs. J. Anim. Sci. Biotechnol..

[59] Liu J., He J., Yang Y., Yu J., Mao X., Yu B., Chen D. (2014). Effects of intrauterine growth retardation and postnatal high-fat diet on hepatic inflammatory response in pigs. Arch. Anim. Nutr..

[60] Olszewski J., Zabielski R., Skrzypek T., Matyba P., Wierzbicka M., Adamski A., Grzesiuk E., Sady M., Gajewski Z., Ferenc K. (2021). Differences in intestinal barrier development between intrauterine growth restricted and normal birth weight piglets. Animals.

[61] Dong L., Zhong X., Ahmad H., Li W., Wang Y., Zhang L., Wang T. (2014). Intrauterine growth restriction impairs small intestinal mucosal immunity in neonatal piglets. J. Histochem. Cytochem..

[62] Bauer R., Walter B., Brust P., Füchtner F., Zwiener U. (2003). Impact of asymmetric intrauterine growth restriction on organ function in newborn piglets. Eur. J. Obstet. Gynecol. Reprod. Biol..

[63] Niu Y., Zhao Y., He J., Yun Y., Shen M., Gan Z., Zhang L., Wang T. (2021). Dietary dihydroartemisinin supplementation alleviates intestinal inflammatory injury through TLR4/NOD/NF-κB signaling pathway in weaned piglets with intrauterine growth retardation. Anim. Nutr..

[64] Wang J., Zhu P., Zheng X., Ma Z., Cui C., Wu C., Zeng X., Guan W., Chen F. (2022). Altered liver metabolism, mitochondrial function, oxidative status, and inflammatory response in intrauterine growth restriction piglets with different growth patterns before weaning. Metabolites.

[65] Niu Y., He J., Zhao Y., Shen M., Zhang L., Zhong X., Wang C., Wang T. (2019). Effect of curcumin on growth performance, inflammation, insulin level, and lipid metabolism in weaned piglets with IUGR. Animals.

[66] Elmhiri G., Mahmood D.F., Niquet-Leridon C., Jacolot P., Firmin S., Guigand L., Tessier F.J., Larcher T., Abdennebi-Najar L. (2015). Formula-derived advanced glycation end products are involved in the development of long-term inflammation and oxidative stress in kidney of IUGR piglets. Mol. Nutr. Food Res..

[67] Wixey J.A., Lee K.M., Miller S.M., Goasdoue K., Colditz P.B., Tracey Bjorkman S., Chand K.K. (2019). Neuropathology in intrauterine growth restricted newborn piglets is associated with glial activation and proinflammatory status in the brain. J. Neuroinflammation.

[68] Amdi C., Lynegaard J.C., Thymann T., Williams A.R. (2020). Intrauterine growth restriction in piglets alters blood cell counts and impairs cytokine responses in peripheral mononuclear cells 24 days post-partum. Sci. Rep..

[69] Han F., Hu L., Xuan Y., Ding X., Luo Y., Bai S., He S., Zhang K., Che L. (2013). Effects of high nutrient intake on the growth performance, intestinal morphology and immune function of neonatal intra-uterine growth-retarded pigs. Br. J. Nutr..

[70] Choi J., Li W., Schindell B., Ni L., Liu S., Zhao X., Gong J., Nyachoti M., Yang C. (2020). Molecular cloning, tissue distribution and the expression of cystine/glutamate exchanger (xCT, SLC7A11) in different tissues during development in broiler chickens. Anim. Nutr..

[71] Yin J., Ren W., Liu G., Duan J., Yang G., Wu L., Li T., Yin Y. (2013). Birth oxidative stress and the development of an antioxidant system in newborn piglets. Free Radic. Res..

[72] Jomova K., Baros S., Valko M. (2012). Redox active metal-induced oxidative stress in biological systems. Transit. Met. Chem..

[73] Gao H., Chen X., Zhao J., Xue Z., Zhang L., Zhao F., Wang B., Wang L. (2022). Integrative analysis of liver metabolomics and transcriptomics reveals oxidative stress in piglets with intrauterine growth restriction. Biology.

[74] Zhao Y., Niu Y., He J., Zhang L., Wang C., Wang T. (2019). Dietary dihydroartemisinin supplementation attenuates hepatic oxidative damage of weaned piglets with intrauterine growth retardation through the Nrf2/ARE signaling pathway. Animals.

[75] Zhang H., Li Y., Su W., Ying Z., Zhou L., Zhang L., Wang T. (2017). Resveratrol attenuates mitochondrial dysfunction in the liver of intrauterine growth retarded suckling piglets by improving mitochondrial biogenesis and redox status. Mol. Nutr. Food Res..

[76] Zhang H., Li Y., Chen Y., Ying Z., Su W., Zhang T., Dong Y., Htoo J.K., Zhang L., Wang T. (2019). Effects of dietary methionine supplementation on growth performance, intestinal morphology, antioxidant capacity and immune function in intra-uterine growth-retarded suckling piglets. J. Anim. Physiol. Anim. Nutr..

[77] Niu Y., He J., Ahmad H., Shen M., Zhao Y., Gan Z., Zhang L., Zhong X., Wang C., Wang T. (2019). Dietary curcumin supplementation increases antioxidant capacity, upregulates Nrf2 and Hmox1 levels in the liver of piglet model with intrauterine growth retardation. Nutrients.

[78] Wan J., Yu Q., Luo J., Zhang L., Ruan Z. (2022). Effects of ferulic acid on the growth performance, antioxidant capacity, and intestinal development of piglets with intrauterine growth retardation. J. Anim. Sci..

[79] Zhang L., Zhang J., Yan E., He J., Zhong X., Zhang L., Wang C., Wang T. (2020). Dietary supplemented curcumin improves meat quality and antioxidant status of intrauterine growth retardation growing pigs via Nrf2 signal pathway. Animals.

[80] Liu Y., Azad M.A.K., Kong X., Zhu Q., Yu Z. (2022). Dietary bile acids supplementation modulates immune response, antioxidant capacity, glucose, and lipid metabolism in normal and intrauterine growth retardation piglets. Front. Nutr..

[81] Tang X., Xiong K., Li M. (2023). Effects of dietary epidermal growth factor supplementation on liver antioxidant capacity of piglets with intrauterine growth retardation. J. Anim. Sci..

[82] Hu L., Peng X., Han F., Wu F., Chen D., Wu D., Feyera T., Zhang K., Che L. (2020). Effects of birth weight and postnatal nutritional restriction on skeletal muscle development, myofiber maturation, and metabolic status of early-weaned piglets. Animals.

[83] Li B., Li W., Ahmad H., Zhang L., Wang C., Wang T. (2015). Effects of choline on meat quality and intramuscular fat in intrauterine growth retardation pigs. PLoS ONE.

[84] Heyer A., Lebret B. (2007). Compensatory growth response in pigs: Effects on growth performance, composition of weight gain at carcass and muscle levels, and meat quality. J. Anim. Sci..

[85] Matyba P., Florowski T., Dasiewicz K., Ferenc K., Olszewski J., Trela M., Galemba G., Słowiński M., Sady M., Domańska D. (2021). Performance and meat quality of intrauterine growth restricted pigs. Animals.

[86] Hu L., Han F., Chen L., Peng X., Chen D., Wu D., Che L., Zhang K. (2018). High nutrient intake during the early postnatal period accelerates skeletal muscle fiber growth and maturity in intrauterine growth-restricted pigs. Genes Nutr..

[87] Liu J., Chen D., Yao Y., Yu B., Mao X., He J., Huang Z., Zheng P. (2012). Intrauterine growth retardation increases the susceptibility of pigs to high-fat diet-induced mitochondrial dysfunction in skeletal muscle. PLoS ONE.

[88] Cheng K., Yu C., Li Z., Li S., Yan E., Song Z., Zhang H., Zhang L., Wang T. (2020). Resveratrol improves meat quality, muscular antioxidant capacity, lipid metabolism and fiber type composition of intrauterine growth retarded pigs. Meat Sci..

[89] He W., Posey E.A., Steele C.C., Savell J.W., Bazer F.W., Wu G. (2023). Dietary glycine supplementation enhances postweaning growth and meat quality of pigs with intrauterine growth restriction. J. Anim. Sci..

